# Ultra-high frequency ultrasound delineated changes in carotid and muscular artery intima-media and adventitia thickness in obese early middle-aged women

**DOI:** 10.1177/14791641221094321

**Published:** 2022-05-30

**Authors:** Johnny KM Sundholm, Linda Litwin, Kristiina Rönö, Saila B Koivusalo, Johan G Eriksson, Taisto Sarkola

**Affiliations:** 1 Children’s Hospital, University of Helsinki and Helsinki University Hospital, Helsinki, Finland; 2 Minerva Foundation Institute for Medical Research, Helsinki, Finland; 3 Department of Congenital Heart Defects and Pediatric Cardiology, SMDZ in Zabrze, SUM, Katowice, Poland; 4 Women’s Hospital, University of Helsinki and Helsinki University Hospital, Helsinki, Finland; 5 Department of Gynecology and Obstetrics, University of Turku and Turku University Hospital, Turku Finland; 6 Department of General Practice and Primary Health Care, University of Helsinki, and Helsinki University Hospital, Helsinki, Finland; 7 Folkhälsan Research Center, Helsinki, Finland; 8 Department of Obstetrics & Gynaecology and Human Potential Translational Research Programme, Yong Loo Lin School of Medicine, National University of Singapore, Singapore; 9 Agency for Science, Technology and Research, Singapore Institute for Clinical Sciences, Singapore, Singapore

**Keywords:** Obesity, arterial stiffness, intima-media thickness, adventitia thickness, ultra-high frequency ultrasound

## Abstract

Obesity is linked to increased arterial size, carotid intima-media thickness and arterial stiffness. The effects of obesity and body composition on muscular artery intima-media and adventitia thickness has previously not been established. The aim of this study was to explore associations between carotid and muscular artery wall layer thickness with body composition and cardiovascular risk factors in early middle-aged women. This is a cross-sectional study including 199 women aged 40±4 years. Arterial lumen (LD), intima-media (IMT) and adventitia thickness (AT) were measured from carotid, brachial and radial arteries using ultra-high frequency ultrasound (22-71 MHz). Women with obesity had increased IMT in carotid (0.47 vs 0.45 mm), brachial (0.19 vs 0.17 mm) and radial arteries (0.16 vs 0.15 mm) and increased brachial AT (0.14 vs 0.13 mm). In multiple regression models all arterial LD (β-range 0.02-0.03 mm/kg/m^2^), IMT (β-range 0.91-3.37 µm/kg/m^2^), AT (β-range 0.73-1.38 µm/kg/m^2^) were significantly associated with BMI. The IMT of all arteries were significantly associated with systolic blood pressure (β-range 0.36-0.85 µm/mmHg), attenuating the association between IMT and BMI (β-range 0.18-2.24 µm/kg/m^2^). Obese early middle-aged women have increased arterial intima media thickness and brachial artery adventitia thickness compared to non-obese counterparts. The association between BMI and intima-media thickness is partly mediated through blood pressure levels.

## Introduction

The prevalence of obesity is increasing worldwide.^1^ Obesity affects the vasculature adversely and is linked to hypertension, insulin resistance and dyslipidemia, all related to adverse cardiovascular outcomes.^2^

Obesity is defined according to WHO as a body mass index (BMI) ⩾30 kgm^2^. BMI does not address fat distribution, with abdominal adiposity being more strongly linked to adverse outcomes than BMI.^3^ The increase in body weight in obesity is not only attributed to fat mass, but there is also simultaneously an increase in lean body mass.^4^

Carotid intima-media thickness (IMT) and different measures of arterial stiffness are commonly used surrogate markers for cardiovascular disease and independent predictors of cardiovascular morbidity.^5,6^ Arterial morphology is altered in obesity; larger arterial lumen diameters (LD) and thicker arterial walls are described independent of other cardiovascular risk factors.^7,8^ Very little is known about artery intima or adventitia layer thickness (AT) in clinical obesity, but obesity has been linked to increased adventitia fibrosis and inflammation in animal models.^9^

Non-invasive ultra-high frequency ultrasound (25-70 MHz) provides the opportunity to assess intima, combined IMT, and adventitia artery layer thickness in superficial peripheral arteries such as in the radial and brachial artery.^10^ Although the relevance of peripheral artery IMT is still somewhat unclear, a few studies link this measure with cardiovascular risk.^11,12^ Studies relating peripheral intima and adventitia layer thickness measurements with artery disease progression are very scarce.^13^

Previous studies have mainly focused on effects of obesity on carotid and femoral artery IMT and arterial stiffness.^14^ There is, to our knowledge, no previous study that has addressed the independent effects of adiposity, abdominal adiposity, and body composition on peripheral artery wall layer thickness.

The aim of this study was to assess how arterial structure and function in carotid and peripheral arteries are related to body size and composition in obesity, and how these associations relate to cardiovascular risk factors in early middle-aged women.

## Patients and methods

This is a cross-sectional study derived from the RADIEL-study, the Finnish Gestational Diabetes Prevention Study. The original cohort has been described earlier, consisting of 728 women with obesity (BMI ⩾30kg/m^2^) or previous gestational diabetes (GDM) planning pregnancy or in the first half of gestation.^15^ Exclusion criteria were age <18 years, multiple pregnancy, pregestational diabetes, regular medication affecting glucose metabolism, physical disability, severe psychiatric disorder, current substance abuse and difficulty cooperating due to inadequate language skills.

We recruited 201 women for cardiovascular assessment approximately 6 ± 0.5 years postpartum between June 2015 and May 2017. Two participants with missing BMI information were excluded from analysis. Prior to pregnancy 70 participants had a history of GDM only, 99 participants had pre-pregnancy obesity only, and 30 participants had both GDM and obesity. At follow-up, 28 participants had a BMI <25, 50 were overweight, and 121 were obese.

The research protocol for the six-year follow-up was approved by the Helsinki University Hospital Ethics Committee for gynecology and obstetrics, pediatrics, and psychiatry (20/13/03/03/2015) with informed written consent obtained from all participants at follow-up.

### Background information

Maternal background information was obtained using a structured questionnaire including information on previous medical diagnoses, present medication, current and previous smoking.

### Anthropometrics, blood pressure and blood work

Height and weight were measured with electronic devices (Seca GmbH & Co. KG, Germany) to the nearest 0.1 cm and 0.1 kg. Hip and waist circumferences were measured using a tape measure to the closest 0.5cm. Abdominal adiposity was defined as a waist-to-hip ratio >0.85. Body composition was assessed using bioelectrical impedance (InBody 720, Indbody Bldg, South Korea).

Blood pressure was measured using oscillometry (Omron Healthcare Europe B.V., The Netherlands) from the right arm in sitting position, after one-hour rest. Hypertension was defined as a resting systolic blood pressure ⩾140 mmHg, a diastolic blood pressure ⩾90 mmHg, or daily antihypertensive medication treatment.

Fasting blood samples were taken for assessment of plasma glucose, insulin, total cholesterol, low-density lipoprotein (LDL), high-density lipoprotein (HDL), triglycerides, high sensitivity C-reactive protein (hsCRP), and glycated hemoglobin (HbA_1c_). Diabetes was defined as fasting glucose ≥7.0 mmol/l, HbA1c ≥ 48 mmol/mol, or daily glucose lowering medication. Homeostatic model assessment for insulin resistance (HOMA-IR) was calculated as fasting glucose (mmol/l)*serum insulin (mIU/l)/22.5.^16^ Hypercholesterolemia was defined as total cholesterol >5.0 mmol/l, low density lipoprotein >3.0 mmol/l, or daily lipid-lowering medication.

Metabolic syndrome was classified according to NCEP ATP III criteria, requiring at least three out of five criteria: 1. Waist circumference >88 cm, 2. Fasting glucose ⩾5.6 mmo/l or glucose lowering medication, 3. triglycerides ⩾ 1.7 mmol/l or lipid lowering treatment, 4. HDL <1.3 mmol/ or lipid lowering treatment 5. Systolic blood pressure ⩾130 mmHg or diastolic BP ⩾85 mmHg, or antihypertensive treatment.^17^

### Vascular ultrasound

Ultra-high frequency ultrasound images of bilateral carotid, and right brachial and radial arteries were obtained by one skilled investigator (TS) using 25, 35 MHz and 55 MHz transducers with the Vevo 770 system for the first 149 participants, and using the corresponding UHF22, UHF48 and UHF70 transducers with the Vevo MD system (VisualSonics, Canada) for the remaining participants.

The common carotid artery was imaged 1 cm proximal to the carotid bulb, the brachial artery imaged 5cm proximal to the cubital fold, and the radial artery 1-2 cm proximal to the palma manus. Cine loops were obtained using highest frequency able to visualize the far wall. Images were analyzed offline by one experienced observer (JKMS) blinded to participant characteristics. Vascular lumen and wall dimensions, including IMT and AT, were measured in end-diastole using electronic calipers as a mean of three measures using the leading-leading edge technique.^10^ Images of radial arteries were assessed for four-line pattern consistent with intima thickness >0.06 mm as previously validated by the authors.^18^ We have previously reported intra and inter-observer agreement Cohen’s Kappa as κ = 1.00 and κ = 0.85 respectively.^13^

Carotid artery lumen diameter (LD) was further measured in peak systole to assess local artery stiffness. The distensibility coefficient and β-stiffness index of the carotid artery was calculated as follows:

$$\mathrm{Carotid}\;\mathrm{artery}\;\mathrm{distensibility}\;\mathrm{coefficient}=100\;*\frac{\left(CCALAS-\;CCALAD\right)/CCALAD}{\left(SBP-DBP\right)}$$

$$\mathrm{Carotid}\;\mathrm{artery}\;\beta -\mathrm{stiffness}\;\mathrm{index}=\frac{\ln\left(\frac{SBP}{DBP}\right)}{\left(CCALDS-CCALDD\right)/CCALDD}$$

Where CCALAS and CCALAD are common carotid artery lumen area in systole and diastole respectively, CCALDS and CCALDD are common carotid artery lumen diameter in systole and diastole respectively, and SBP and DBP are systolic and diastolic blood pressures.

Carotid arteries were screened for plaques from the proximal common carotids, throughout the bifurcation and the proximal parts of internal and external carotids using a 12 MHz linear transducer (Vivid 7, GE, MA USA). Plaques were defined as a focal increase in vascular wall thickness exceeding 0.5 mm or more than 50% of the surrounding IMT, or as a focal vascular wall thickness exceeding 1.5 mm.^19^ Repeat measurements to assess intra- and inter-observer variabilities were independently performed on a subset of images (N = 40) by primary observer (JKMS) and a second observer (TS) giving Cohen’s Kappa values κ = 0.89 and κ = 0.83 respectively.

### Pulse wave velocity

Central (carotid-femoral; cfPWV) and peripheral (carotid-radial; crPWV) pulse wave velocities were measured using mechanosensors (Complior Analyse, Alam Medical, Saint-Quentin-Fallavier, France) in supine position. Transit time was recorded using sensors and transit distance measured using a tape measure to the closest 0.1 cm. Carotid-femoral distance was multiplied by a 0.8. Sensors were set on right carotid, radial and femoral arteries to assess transit times. At least two recordings were obtained, and a third recording performed in the setting of a difference of at least 0.5 m/s (10%) between parallel measurements. The mean of two measurements with the lowest tolerance values were used in final analysis.

### Data analysis

Continuous variables are presented as median and 25-75th percentiles. Dichotomous variables are presented as frequencies and percentages. The cohort was stratified in groups in relation to BMI with normal weight and overweight participants combined into one group. Group-wise comparison was done using Mann-Whitney U-test.

Univariate associations were assessed using Spearman rank correlations. Multiple linear regression was used to assess the independent associations between different measures of adiposity, body sizes and other cardiovascular risk parameters, and vascular measures. The first model included age and BMI as independent predictors to assess associations between obesity and the vascular profile. The second model included age, lean body mass and body fat percentage as independent predictors to assess relations between adiposity and vascular variables, whereas the third model included age, BMI and increased waist-to-hip ratio (⩾0.85) to assess associations between abdominal adiposity and vascular variables. Further multiple linear regression models adjusted for age and BMI were used to assess the independent association of cardiovascular risk factors on vascular variables. As previous GDM influenced baseline BMI, it was included in all models to avoid confounding. Multicollinearity of independent variables were assessed using variance influence factor, with a VIF<2.5 deemed appropriate. Normality and homoscedasticity of residuals were assessed. All tests were two-tailed and a p-value<0.05 deemed significant. Statistical analysis was performed using SPSS v.24 (IBM, NY, USA) and StataMP v.15.1 (Stata Corp., TX, USA).

## Results

Background characteristics of the study population are presented in Table 1. The obese participants had increased weight, waist-to-hip ratio, fat percentage and lean body mass compared to non-obese participants with no difference in age and height. Obese participants had higher blood pressure, increased prevalence of hypertension, lower HDL, higher triglycerides, higher hsCRP, and higher prevalence of metabolic syndrome compared to non-obese participants, but with no significant difference in type 2 diabetes prevalence and LDL concentration.

**Table 1.** Background characteristics of study population.

	BMI <30 (n = 78)	BMI ⩾30 (n = 121)	p-value
	Median (IQR); N (%)	Median (IQR); N (%)	
Age [years]	40.8 (38.0–43.0)	40.3 (37.9–44.1)	0.853
Parity	2 (1–3)	1 (1–2)	0.229
Height [cm]	167 (162–171)	166 (162–170)	0.427
Weight [kg]	71.5 (63.6–80.0)	97.3 (88.9–109.8)	<0.001
Body mass index [kg/m2]	26.2 (23.9–27.7)	35.3 (33.3–38.3)	<0.001
Waist-hip ratio	0.82 (0.79–0.86)	0.89 (0.84–0.93)	<0.001
Lean body mass [kg}	42.0 (38.2–44.9)	49.0 (44.7–53.0)	<0.001
Body fat percentage [%]	42.0 (39.0–43.6)	50.5 (48.8–52.1)	<0.001
Systolic blood pressure [mmHg]	112 (106–118)	120 (111–132)	<0.001
Diastolic blood pressure [mmHg]	66 (61–72)	69 (65–76)	0.003
Hypertension medication [y/n]	2 (3%)	16 (13%)	0.011
Hypertension [y/n]^a^	2 (3%)	25 (21%)	<0.001
Fasting plasma glucose [mmol/l]	5.0 (4.7–5.4)	5.2 (4.8–5.6)	0.161
HbA1c [mmol/mol]	35 (33–37)	36 (34–39)	0.092
Glucose-lowering medication [y/n]	2 (3%)	8 (7%)	0.321
HOMA-IR	1.37 (1.06–2.12)	2.92 (1.84–4.16)	<0.001
Type 2 diabetes [y/n]^b^	2 (3%)	9 (7%)	0.207
Low-density lipoprotein [mmol/l]	2.9 (2.4–3.4)	2.9 (2.5–3.5)	0.840
High-density lipoprotein [mmol/l]	1.61 (1.42–1.88)	1.32 (1.13–1.56)	<0.001
Triglycerides [mmol/l]	0.65 (0.55–0.94)	0.93 (0.71–1.51)	<0.001
Lipid-lowering medication [y/n]	2 (3%)	2 (2%)	0.646
Dyslipidemia [y/n]^c^	32 (41%)	49 (40%)	0.883
High-sensitivity CRP [mg/l]	0.6 (0.3–1.3)	2.6 (1.2–4.4)	<0.001
Metabolic syndrome [y/n]^d^	3 (4%)	43 (36%)	<0.001
History of smoking [y/n]	43 (55%)	61 (50%)	0.562
Smoking >10 pack years [y/n]	13 (17%)	24 (20%)	0.709
Family history of CVD [y/n]	52 (67%)	71 (59%)	0.363
Previous GDM [y/n]^e^	66 (85%)	63 (52%)	<0.001

CVD–cardiovascular disease, GDM–gestational diabetes.

a Hypertension was defined as systolic blood pressure ⩾140 mmHg, diastolic blood pressure ⩾90 mmHg, or daily antihypertensive medication.

b Diabetes was defined as fasting glucose ⩾7.0 mmol/l, HbA1c ⩾48 mmol/mol, or daily glucose lowering medication.

c Dyslipidemia was defined as total cholesterol ⩾5.0 mmol/l, low-density lipoprotein ⩾3.0 mmol/l, or daily lipid-lowering medication.

d Metabolic syndrome was defined according to NCEP ATP III criteria.

e GDM in any previous pregnancies.

**Figure 1.**
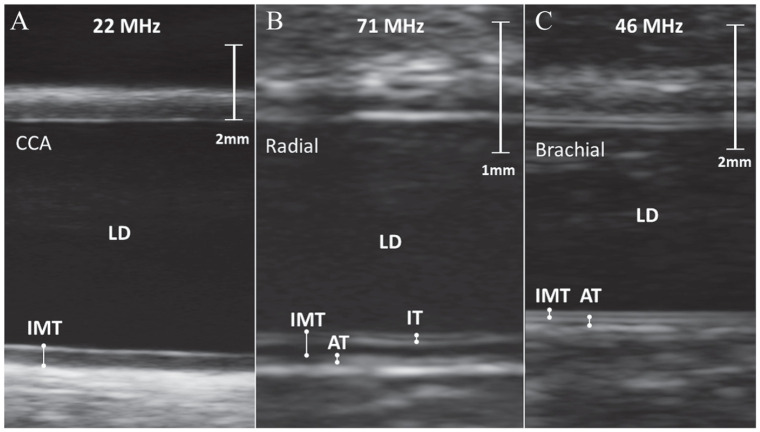
Sample images obtained with ultra-high frequency ultrasound of A) a common carotid artery, B) a radial artery with 4-line intimal thickening and C) a brachial artery. AT–adventitia thickness; CCA–common carotid artery; IMT–intima-media thickness; IT–intima-thickness; LD–lumen diameter.

Group-wise comparisons of vascular parameters are presented in Table 2 and sample images in Figure 1. Obese participants presented with increased lumen diameter and intima-media thickness in carotid, brachial and radial arteries. Brachial artery adventitia thickness was increased in obese participants compared with non-obese. There was no difference in plaque presence, presence of multiple plaques, plaque size, or prevalence of radial artery intima thickening between the groups.

**Table 2:** . Vascular outcomes stratified for non-obese and obese participants.

	BMI <30 (n = 78)	BMI ⩾30 (n = 121)	p-value
	Median (IQR); N (%)	Median (IQR); N (%)	
Carotid Artery			
Lumen diameter [mm]	5.30 (4.98–5.53)	5.47 (5.21–5.77)	<0.001
Intima-media thickness [mm]	0.45 (0.41–0.50)	0.47 (0.44–0.53)	0.011
Plaques [y/n]	17 (22%)	15 (12%)	0.109
Multiple plaques [y/n]	6 (8%)	3 (2%)	0.157
Total plaque area [mm2]^a^	0.13 (0.06–0.25)	0.10 (0.06–0.13)	0.261
Brachial artery			
Lumen diameter [mm]	3.15 (2.93–3.29)	3.25 (3.08–3.53)	0.042
Intima-media thickness [mm]	0.17 (0.15–0.18)	0.19 (0.17–0.21)	0.002
Adventitia thickness [mm]	0.13 (0.11–0.14)	0.14 (0.12–0.16)	0.003
Radial artery			
Lumen diameter [mm]	1.91 (1.70–2.07)	1.95 (1.81–2.14)	0.001
Intima media thickness [mm]	0.15 (0.13–0.16)	0.16 (0.14–0.18)	<0.001
Adventitia thickness [mm]	0.08 (0.07–0.09)	0.08 (0.07–0.09)	0.124
Intima thickening (>0.06 mm)	5 (6%)	4 (3%)	0.313
Arterial stiffness			
Carotid-femoral PWV [m/s]	7.0 (6.5–7.8)	7.5 (6.8–8.4)	0.002
Carotid-radial PWV [m/s]	9.2 (8.6–10.2)	9.1 (8.3–9.9)	0.222
Carotid Beta stiffness index	3.2 (2.6–3.9)	3.2 (2.3–4.1)	0.372
Carotid Distensibility Coeffcient [%/10 mmHg]	3.7 (3.0–4.6)	3.3 (2.5–4.3)	0.045

a Among participants with plaques.

PWV–Pulse wave velocity.

Central carotid-femoral pulse wave velocity was higher and carotid distensibility coefficient was lower among obese participants, but no differences were seen in carotid-radial PWV or carotid β-stiffness between groups.

Univariate associations between vascular dimensions, vascular stiffness measures and different adiposity and anthropometric measures are presented in Supplemental table 1. Only carotid LD correlated with participants height (Spearman’s ρ = 0.21, p = 0.003). All vascular dimensions were to different extents significantly correlated to BMI, lean body mass and body fat% (ρ ranging 0.16-0.37), except radial LD which only correlated with BMI and body fat%, not with lean body mass (ρ = 0.06, p = 0.411). Waist-to-hip ratio correlated with carotid LD (ρ = 0.19, p = 0.009), radial LD (ρ = 0.15, p = 0.037) and IMT (ρ = 0.24, p = 0.001), as well as Brachial LD (ρ = 0.23, p = 0.001), IMT (ρ = 0.23, p = 0.002) and AT (ρ = 0.17, p = 0.02).

Carotid β-stiffness index was not significantly correlated with any anthropometric measures (ρ ranging 0.08-0.10, p-values ns.), whereas carotid distensibility coefficient correlated negatively with BMI (ρ = −0.15, p = 0.048), and body fat% (ρ = −0.18, p = 0.023) but not with lean body mass or waist-to-hip ratio. Carotid-femoral PWV was positively associated with BMI (ρ = 0.21, p = 0.004), lean body mass (ρ = 0.20, p = 0.005), body fat% (ρ = 0.17, p = 0.017) and waist-to-hip ratio (ρ = 0.28, *p* < 0.001), whereas carotid-radial PWV was negatively associated with BMI (ρ=-0.18, p=0.012) and body fat% (ρ=-0.20, p=0.007), but not significantly correlated with lean body mass or waist-to-hip ratio.

In multiple regression models assessing independent associations of BMI on vascular parameters adjusted for age and previous GDM (Table 3), BMI was independently associated with all arterial dimensions as well as carotid-femoral PWV, but not with carotid distensibility coefficient, β-stiffness index, or carotid-radial PWV. All arterial IMT and all measures of arterial stiffness except carotid β-stiffness index were associated with age. There was no independent effect of age on arterial LD and AT.

**Table 3.** Multiple linear regression models showing associations of age and BMI with vascular parameters.

Dependent variable	N	Adjusted R2	Model P-value
Independent variables	B	CI95	P-value
Carotid LD [mm]	176	0.09	<0.001
Constant	4.76	4.09;5.44	<0.001
Age [years]	−0.01	−0.02;0.01	0.480
BMI [kg/m2]	0.03	0.01;0.04	<0.001
Carotid IMT [µm]	177	0.19	<0.001
Constant	133.1	29.6;236.7	0.012
Age [years]	5.60	3.38;7.81	<0.001
BMI [kg/m2]	3.37	1.59;5.16	<0.001
Brachial LD [mm]	182	0.08	<0.001
Constant	2.77	2.12;3.42	<0.001
Age [years]	−0.01	−0.02;0.00	0.190
BMI [kg/m2]	0.02	0.01;0.03	<0.001
Brachial IMT [µm]	179	0.21	<0.001
Constant	45.1	3.4;86.7	0.034
Age [years]	2.12	1.26;2.99	<0.001
BMI [kg/m2]	1.63	0.93;2.34	<0.001
Brachial AT [µm]	175	0.08	<0.001
Constant	57.5	12.3;102.8	0.013
Age [years]	0.77	−0,17;1.71	0.106
BMI [kg/m2]	1.38	0.62;2.14	<0.001
Radial LD [mm]	179	0.06	0.003
Constant	1.47	1.01;1.93	<0.001
Age [years]	−0.00	−0.01;0.01	0.760
BMI [kg/m2]	0.02	0.01;0.02	<0.001
Radial IMT [µm]	179	0.13	<0.001
Constant	39.7	−4.5;83.9	0.078
Age [years]	2.20	1.28;3.12	<0.001
BMI [kg/m2]	0.91	0.16;1.66	0.018
Radial AT [µm]	176	0.10	<0.001
Constant	15.4	−11.9;42.8	0.267
Age [years]	1.00	0.43;1.57	0.001
BMI [kg/m2]	0.73	0.25;0.1.20	0.003
Carotid DC [%/10mmHg]	159	0.05	0.001
Constant	7.15	5.02;9.28	<0.001
Age [years]	−0.06	−0.11;−0.02	0.007
BMI [kg/m2]	−0.03	−0.07;0.00	0.057
Carotid β-stiffness index	159	0.00	0.359
Constant	1.72	−0.08;3.53	0.061
Age [years]	0.03	−0.01;0.06	0.168
BMI [kg/m2]	0.02	−0.01;0.05	0.272
Carotid-Femoral PWV [m/s]	189	0.10	<0.001
Constant	3.02	1.08;4.95	0.002
Age [years]	0.08	0.04;0.12	<0.001
BMI [kg/m2]	0.04	0.00;0.07	0.025
Carotid-Radial PWV [m/s]	189	0.06	0.003
Constant	7.15	4.67;9.63	<0.001
Age [years]	0.08	0.03;0.13	0.002
BMI [kg/m2]	−0.03	−0.08;0.01	0.116

All models are adjusted for previous GDM. AT–Adventitia thickness; BMI–Body mass index; DC–Distensibility coefficient; IMT–Intima media thickness; LD–-Lumen diameter; PWV–Pulse wave velocity.

In multiple regression models assessing associations between body fat percentage and vascular parameters adjusting for lean body mass, age and previous GDM (Table 4.), there was no significant independent association between body fat% and carotid LD or IMT, or radial IMT, which were predicted by lean body mass only. Brachial LD and IMT were independently predicted by body fat% and lean body mass, whereas brachial adventitia thickness and radial LD were predicted only by body fat%. There were no significant independent associations between body fat% or lean body mass and radial AT, cfPWV, and local carotid artery stiffness measures. Carotid-radial PWV was negatively associated with body fat%.

**Table 4.** Multiple linear regression models showing the independent associations between lean body mass and fat percentage and vascular outcomes.

Dependent variable	N	Adjusted R2	Model P-value
Independent variables	B	CI95	P-value
Carotid LD [mm]	175	0.13	<0.001
Constant	4.00	3.17;4.83	<0.001
Age [years]	−0.01	−0.02;0.01	0.452
Lean body mass [kg]	0.02	0.01;0.03	<0.001
Body fat percentage [%]	0.01	0.00;0.02	0.088
Carotid IMT [µm]	176	0.17	<0.001
Constant	68.8	−63.6;201.2	0.307
Age [years]	5.64	3.39;7.88	<0.001
Lean body mass [kg]	1.73	0.02;3.44	0.048
Body fat percentage [%]	1.96	−0.17;4.09	0.071
Brachial LD [mm]	181	0.09	<0.001
Constant	2.14	1.32;2.97	<0.001
Age [years]	−0.01	−0.02;0.00	0.180
Lean body mass [kg]	0.01	0.00;0.02	0.009
Body fat percentage [%]	0.02	0.00;0.03	0.017
Brachial IMT [µm]	178	0.23	<0.001
Constant	−0.63	−53.1;51.8	0.981
Age [years]	2.13	1.27;2.98	<0.001
Lean body mass [kg]	1.09	0.45;1.73	0.001
Body fat percentage [%]	0.99	0.17;1.83	0.019
Brachial AT [µm]	174	0.06	0.007
Constant	33.7	−24.3;91.8	0.253
Age [years]	0.76	−0.19;1.71	0.116
Lean body mass [kg]	0.52	−0.18;1.22	0.143
Body fat percentage [%]	0.95	0.05;1.84	0.038
Radial LD [mm]	178	0.07	0.01
Constant	1.18	0.60;1.77	<0.001
Age [years]	0.00	−0.01;0.01	0.718
Lean body mass [kg]	0.00	−0.01;0.01	0.767
Body fat percentage [%]	0.02	0.01;0.02	0.001
Radial IMT [µm]	178	0.14	<0.001
Constant	15.8	−40.5;72.0	0.580
Age [years]	2.21	1.28;3.13	<0.001
Lean body mass [kg]	0.79	0.10;1.84	0.025
Body fat percentage [%]	0.34	−0.56;1.22	0.459
Radial AT [µm]	175	0.09	<0.001
Constant	0.15	−34.8;35.0	0.993
Age [years]	1.01	0.43;1.58	0.001
Lean body mass [kg]	0.34	−0.01;0.77	0.122
Body fat percentage [%]	0.49	−0.07;1.05	0.089
Carotid DC [%/10mmHg]	158	0.05	0.024
Constant	7.88	5.21;10.6	<0.001
Age [years]	−0.06	−0.11;−0.02	0.008
Lean body mass [kg]	−0.01	−0.04;0.03	0.667
Body fat percentage [%]	−0.03	−0.07;0.01	0.127
Carotid β-stiffness index	158	0.02	0.518
Constant	1.33	−0.91;3.57	0.243
Age [years]	0.03	−0.01;0.06	0.179
Lean body mass [kg]	0.01	−0.02;0.04	0.482
Body fat percentage [%]	0.01	−0.02;0.04	0.564
Carotid-Femoral PWV [m/s]	188	0.10	<0.001
Constant	1.79	−0.66;4.25	0.151
Age [years]	0.08	0.04;0.12	<0.001
Lean body mass [kg]	0.03	0.00;0.06	0.064
Body fat percentage [%]	0.02	−0.02;0.06	0.239
Carotid-Radial PWV [m/s]	188	0.06	0.003
Constant	7.63	4.48;10.8	<0.001
Age [years]	0.08	0.03;0.13	0.002
Lean body mass [kg]	0.02	−0.02;0.06	0.290
Body fat percentage [%]	−0.06	−0.11;-0.01	0.030

All models are adjusted for previous GDM. AT–Adventitia thickness; DC–Distensibility coefficient; IMT–Intima media thickness; LD–Lumen diameter; PWV–Pulse wave velocity.

In multiple regression models assessing associations between abdominal adiposity (WHR⩾0.85 and BMI) and vascular parameters adjusting for age and previous GDM (Supplemental table 2), there was an independent association between increased waist-to-hip ratio (>0.85) and carotid-femoral PWV (β=−0.72 m/s, CI95% 0.32;1.11, *p* < 0.001) only.

When assessing how obesity related cardiovascular risk factors including systolic blood pressure, hypertension medication, LDL cholesterol, triglycerides, glycemic measures, and hsCRP were associated with arterial dimensions, none of the cardiovascular risk factors predicted arterial LD or arterial AT. The IMT of all arteries were significantly associated with systolic blood pressure (carotid: β = 0.85 µm/mmHg, CI95% 0.15;1.55, p = 0.017, brachial: β = 0.36 µm/mmHg, CI95% 0.08;0.63, p = 0.011, radial: β = 0.50 µm/mmHg, CI95% 0.21;0.78, p = 0.001). Furthermore, brachial and radial artery IMT were higher among participants with hypertension medication (y/n, brachial: β = 16.8, CI95% 3.78;29.9, p = 0.012, radial: β = 19.0 CI95% 5.4;32.5, p = 0.006). Inclusion of systolic blood pressure and hypertensive treatment in the regression model attenuated the association between IMT and BMI (carotid: β = 2.24 µm/kg/m^2^, CI95% 0.37.;4.12, p = 0.019, brachial: β = 1.08 µm/kg/m^2^, CI95% 0.35;1.82, p = 0.004, radial: β = 0.18 µm/kg/m^2^, CI95% -0.50;0.86, p = 0.609, supplemental table 3).

Both carotid distensibility coefficient and carotid β-stiffness index were predicted by metabolic syndrome (y/n, DC: β = −0.80%/10mmHg, CI95% -1.27;−0.33, p=0.002, BSI: β =0.50 m, CI95% 0.09;0.90, p = 0.015) and related with >10 pack years of regular cigarette smoking (y/n, DC: β = −0.49%/10mmHg, CI95% -0.95;−0.04, p = 0.035, BSI: β = 0.42 m, CI95% 0.03;0.81, p = 0.036, supplemental table 3.). The significant association between central arterial stiffness (cfPWV) and BMI disappeared (β = −0.02 m/s/kg/m^2^, CI95% -0.05;0.02, p = 0.384) after inclusion of WHR > 0.85 (β = 0.61 m/s CI95% 0.23;1.00, p = 0.002), triglycerides (β = 0.48 m/s/mmol/l CI95% 0.17;0.80, p = 0.003), and hypertension (y/n, β = 0.62 m/s, CI95% 0.10;1.13, p = 0.018) in the model (supplemental table 3.).

Plaque presence, plaque area, plaque number and radial artery intimal thickening were not associated with any anthropometric measures or cardiovascular risk factors in our sample.

## Discussion

This study assessed associations between obesity, adiposity, cardiovascular risk parameters and arterial structure and stiffness in early middle-aged women. Arterial lumen diameters, intima-media and adventitia layers were increased in obese women compared to non-obese women. Increased arterial layers were related with both increased lean body mass, body fat percentage, and blood pressure among the obese women. Central aortic stiffness was increased in obese women and related with abdominal adiposity, systolic blood pressure, and increased blood triglyceride concentration.

Artery sizes were associated with BMI, but not with CVD risk factors or age. An increased carotid, radial and brachial artery LD has previously been reported in obesity and further shown to be reversible with weight loss.^20,21^ Brachial artery LD has been reported to be associated with lean body mass in non-obese, whereas radial LD has been related with wrist circumference, suggesting that arterial size is dependent on end-organ size and volumetric blood flow.^22,23^

We found significant linear associations between BMI and IMT in all arteries studied. Arterial IMT did not correlate with LD suggesting a different pathophysiological mechanism for this BMI related vascular wall remodeling. Associations between BMI and IMT in all arteries were attenuated after adjustment for systolic blood pressure suggesting that the adverse effect of obesity on the vascular wall thickness is partly mediated by blood pressure as previously shown.^24^

Both brachial and radial artery adventitia thicknesses were associated with BMI in our sample. We were unable to find any previous human adult studies addressing associations between artery adventitia layer thickness and body size or CVD risk factors. Emerging evidence from preclinical and animal studies suggest a role of the adventitia in the atherogenic process and adventitia remodeling as a part of the arterial pathophysiologic spectrum.^25^

Increased waist-to-hip ratio was only associated with cfPWV after adjustment for BMI. This suggests that the adverse effect of abdominal adiposity mainly influences central aortic stiffness with less apparent influence on vascular morphology. This is in line with previous studies showing significant associations between central arterial stiffness, carotid IMT and abdominal adiposity, and with the association between carotid IMT and abdominal adiposity disappearing after adjusting for BMI.^26,27^

Vascular wall layers were unrelated to lipid profiles, glycemic measures, and hsCRP in our study. Carotid IMT has previously been shown to correlate with LDL in patients with familiar hypercholesterolemia and in men, but this relation is not established in women.^28^ We were unable to find any association between carotid IMT and any measures of glycemic control as previously reported.^29^ This might be related to the low prevalence of diabetes in our sample. Although hsCRP was increased among our obese subjects, we could not demonstrate a mediating effect of low-grade inflammation on arterial parameters.

We were unable to find associations between BMI or abdominal adiposity and local arterial stiffness. Both carotid distensibility and β-stiffness index were associated with history of regular smoking and metabolic syndrome. Previous studies have shown a significant decrease in carotid distensibility associated with BMI, but they calculated distensibility using lumen diameter, not area, leading to a negative association between distensibility and lumen size.^14^

Central arterial stiffness (cfPWV) was associated with BMI in our sample. This association disappeared when adjusted for waist-to-hip ratio, hypertension, and triglyceride concentrations suggesting that the adverse effect of obesity could be mediated through abdominal adiposity, blood pressure and triglyceride concentrations. Our results are then similar to a recent meta-analysis, where blood pressure was the only predictor of central arterial stiffness, with no independent effect of obesity.^30^ Peripheral arterial stiffness (crPWV) was only inversely associated with body fat% with no further associations observed. The role of crPWV as a surrogate marker for CVD is not well established and the significance of this finding remains unclear.^31^

Surprisingly we found no significant predictors of plaque presence and radial artery intimal thickening in our sample. This might be related to the low prevalence of these vascular outcomes in this relatively young population.

**Figure 2.**
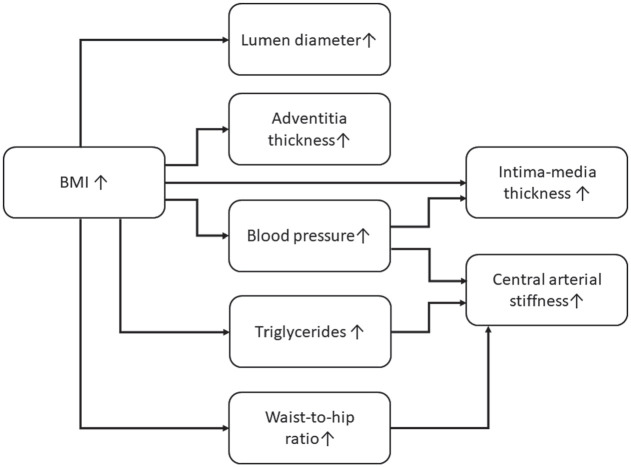
The effects of BMI on arterial size, arterial intima-media thickness, adventitia thickness and arterial stiffness. Whereas arterial lumen size and adventitia thickness is mainly related to body size the increase in intima-media thickness is partly derived through increased blood pressure levels. Central arterial stiffness is mainly affected through increased blood pressure, increased waist-to-hip ratio, and an increase in triglyceride concentrations.

Taken together, this study shows a link between obesity and increased lumen diameter, intima-media thickness, adventitia thickness and arterial stiffness. Our findings suggest that the changes appear through different pathophysiological trajectories (Figure 2.). Whereas arterial lumen size and adventitia thickness are mainly related to body size, the increase in intima-media thickness is partly derived through increased blood pressure in obesity. Central arterial stiffness seems mainly predicted by increased blood pressure, increased waist-to-hip ratio, and an increase in triglyceride concentrations among early middle-aged women with obesity.

### Study limitations

The cross-sectional study design limits inferring causality in the associations observed. The extensively assessed cardiovascular surrogate markers allows us to describe a marked heterogeneity of the associations between body composition and cardiovascular risk factors and vascular outcomes. Women without previous GDM or obesity were excluded in the primary trial and thus the cohort did not include a healthy reference. The exclusion of a healthy reference would bias the results towards null, and an underestimated effect is possible. Further, participants with significant hypertension and diabetes at recruitment were excluded, limiting the prevalence and cumulative exposure to these risk factors, which might reduce their effect on vascular measures. Another limitation is the lack of information on parental cardiovascular disease history. The inclusion of women only with high prevalence of obesity and a narrow age range limits the interpretation of the effect of sex and age on outcomes. The results are, however, strengthened by the relatively large sample size, well characterized anthropometrics, cardiovascular risk profile, and background factors.

## Conclusion

In this cross-sectional study, we show that early middle-aged women with obesity have an altered vascular profile with increased arterial size, central arterial stiffness, arterial intima media and adventitia thickness, and decreased carotid distensibility. We further propose that the arterial changes are derived through different pathophysiological mechanisms. Arterial lumen diameter and adventitia thickness are mainly related with BMI. The effect of BMI on arterial intima-media thickness is partly mediated through increased blood pressure levels, whereas the effect on central arterial stiffness is mediated through abdominal adiposity, hypertension and increased triglyceride concentrations.

## Supplemental Material

sj-pdf-1-dvr-10.1177_14791641221094321 – Supplemental material for Ultra-high frequency ultrasound delineated changes in carotid and muscular artery intima-media and adventitia thickness in obese early middle-aged women Click here for additional data file. Supplemental material, sj-pdf-1-dvr-10.1177_14791641221094321 for Ultra-high frequency ultrasound delineated changes in carotid and muscular artery intima-media and adventitia thickness in obese early middle-aged women by Johnny KM Sundholm, Linda Litwin, Kristiina Rönö, Saila B Koivusalo, Johan G Eriksson and Taisto Sarkola in Diabetes & Vascular Disease Research

## Appendix

### Abbreviations

AT Adventitia thickness

BMI Body mass index

cfPWV Carotid-Femoral pulse wave velocity

crPWV Carotid-Radial pulse wave velocity

DC Distensibility coefficient

GDM Gestational diabetes mellitus

HbA1c Glycated hemoglobin

HDL High-density lipoprotein cholesterol

HOMA-IR Homeostatic model assessment for insulin resistance

hsCRP High-sensitivity C-reactive protein

IMT Intima-media thickness

LD Lumen diameter

LDL Low-density lipoprotein cholesterol

PWV Pulse wave velocity
