# Supplementary material for: Ultra-high frequency ultrasound delineated changes in carotid and muscular artery intima-media and adventitia thickness in obese early middle-aged women
Source: Diab Vasc Dis Res. 2022 May 30;19(3):14791641221094321. doi: 10.1177/14791641221094321 (PMC9160911; doi:10.1177/14791641221094321)
Supplement: sj-pdf-1-dvr-10.1177_14791641221094321 – Supplemental material for Ultra-high frequency ultrasound delineated changes in carotid and muscular artery intima-media and adventitia thickness in obese early middle-aged women [file sj-pdf-1-dvr-10.1177_14791641221094321.pdf]

Supplemental Table 1. Univariate correlations between vascular parameters and different measures of body size and composition.

Univariate correlation<br>Spearman's ρ	Height	BMI	LBM	Fat%
Carotid artery				
Lumen diameter	0.21**	0.29***	0.37***	0.22**
Intima-media thickness	0.04	0.22**	0.20**	0.22**
Radial artery				
Lumen diameter	-0.09	0.19**	0.06	0.21**
Intima-media thickness	0.11	0.17*	0.20**	0.16*
Adventitia thickness	0.06	0.18*	0.16*	0.18*
Brachial artery				
Lumen diameter	0.11	0.28***	0.26***	0.26***
Intima-media thickness	0.11	0.31***	0.32***	0.29***
Adventitia thickness	0.08	0.22**	0.22**	0.23**
Arterial Stiffness				
Carotid β-stiffness index	0.08	0.08	0.09	0.10
Carotid distensibility coefficient	-0.08	-0.15*	-0.15	-0.17*
Carotid-femoral PWV	0.06	0.21**	0.20**	0.17*
Carotid-radial PWV	0.04	-0.18*	-0.09	-0.20**

Supplemental Table 1. * - significant at 0.05-level; ** - significant at 0.01-level; *** - significant at 0.001-level. BMI – Body mass index; LBM – Lean body mass; Fat% - Body fat percentage; PWV – Pulse wave velocity.

Supplemental Table 2. Multiple linear regression models assessing independent associations between abdominal adiposity, adjusted for age and BMI, and vascular parameters.

Dependent variable	N	Adjusted R^2	Model P-value
Independent variables	*B*	*CI95*	*P-value*
Carotid Lumen diameter [mm]	174	0.10	<0.001
Constant	*4.87*	*4.19;5.55*	*<0.001*
Age [years]	*-0.00*	*-0.02;0.01*	*0.509*
Body mass index [kg/m²]	***0.02***	***0.01;0.03***	***0.004***
Waist-to-hip ratio > 0.85 [y/n]	*0.13*	*-0.01;0.27*	*0.069*
Carotid intima-media thickness [µm]	175	0.19	<0.001
Constant	*122.3*	*17.2;227.5*	*0.023*
Age [years]	***5.47***	***3.26;7.69***	***<0.001***
Body mass index [kg/m²]	***4.09***	***2.04;6.14***	***<0.001***
Waist-to-hip ratio > 0.85 [y/n]	*-13.34*	*-35.49;8.82*	*0.236*
Brachial Lumen diameter [mm]	180	0.09	<0.001
Constant	*2.83*	*2.17;3.49*	*<0.001*
Age [years]	*-0.01*	*-0.02;0.00*	*0.201*
Body mass index [kg/m²]	***0.02***	***0.01;0.03***	***0.002***
Waist-to-hip ratio > 0.85 [y/n]	*0.08*	*-0.05;0.22*	*0.235*
Brachial intima-media thickness [µm]	177	0.21	*<0.001*
Constant	*46.5*	*4.08;88.8*	*0.032*
Age [years]	***2.11***	***1.24;2.97***	***<0.001***
Body mass index [kg/m²]	***1.56***	***0.76;2.37***	***<0.001***
Waist-to-hip ratio > 0.85 [y/n]	*2.73*	*--6.09;11.54*	*0.542*
Brachial adventitia thickness [µm]	173	0.09	*<0.001*
Constant	*59.8*	*13.87;105.77*	*0.011*
Age [years]	*0.70*	*-0.23;1.64*	*0.138*
Body mass index [kg/m²]	***1.33***	***0.47;2.20***	***0.003***
Waist-to-hip ratio > 0.85 [y/n]	*3.09*	*-6.24;12.41*	*0.514*
Radial Lumen diameter [mm]	177	0.07	0.003
Constant	*1.43*	*0.96;1.89*	*<0.001*
Age [years]	*0.00*	*-0.01;0.01*	*0.688*
Body mass index [kg/m²]	***0.02***	***0.01;0.03***	***<0.001***
Waist-to-hip ratio > 0.85 [y/n]	*-0.05*	*-0.14;0.05*	*0.345*
Radial intima-media thickness [µm]	177	0.13	*<0.001*
Constant	*43.8*	*-1.3;88.9*	*0.57*
Age [years]	***2.21***	***1.28;3.14***	***<0.001***
Body mass index [kg/m²]	*0.70*	*-0.16;1.56*	*0.112*
Waist-to-hip ratio > 0.85 [y/n]	*5.11*	*-4.24;14.46*	*0.282*
Radial adventitia thickness [µm]	174	0.10	*<0.001*
Constant	*12.8*	*-14.9;40.6*	*0.362*
Age [years]	***1.02***	***0.45;1.59***	***0.001***
Body mass index [kg/m²]	***0.82***	***0.29;1.36***	***0.003***
Waist-to-hip ratio > 0.85 [y/n]	*-3.16*	*-8.93;2.60*	*0.281*
Carotid DC [%/10mmHg]	157	0.05	0.013
Constant	*7.00*	*4.84;9.16*	*<0.001*
Age [years]	***-0.06***	***-0.11;-0.02***	***0.007***

Body mass index [kg/m²]	*-0.02*	*-0.06;0.02*	*0.237*
Waist-to-hip ratio > 0.85 [y/n]	*-0.22*	*-0.65;0.21*	*0.312*
Carotid β-stiffness index	157	0.00	0.521
Constant	*1.75*	*-0.09;3.59*	*0.062*
Age [years]	*0.03*	*-0.01;0.07*	*0.164*
Body mass index [kg/m²]	*0.01*	*-0.02;0.05*	*0.422*
Waist-to-hip ratio > 0.85 [y/n]	*0.05*	*-0.32,0.41*	*0.796*
Carotid-Femoral PWV [m/s]	187	0.15	<0.001
Constant	*3.61*	*-1.71;5.51*	*<0.001*
Age [years]	***0.08***	***0.04;0.12***	***<0.001***
Body mass index [kg/m²]	*0.01*	*-0.03;0.04*	*0.675*
Waist-to-hip ratio > 0.85 [y/n]	***0.72***	***0.32;1.11***	***<0.001***
Carotid-Radial PWV [m/s]	187	0.05	0.02
Constant	*7.47*	*4.97;9.96*	*<0.001*
Age [years]	***0.08***	***0.03;0.13***	***0.004***
Body mass index [kg/m²]	*-0.05*	*-0.09;0.00*	*0.061*
Waist-to-hip ratio > 0.85 [y/n]	*0.39*	*--0.12;0.91*	*0.132*

Supplemental table 2. All models are adjusted for previous GDM. BMI – Body mass index; DC – Distensibility coefficient; PWV – Pulse wave velocity.

Supplemental table 3. Multiple linear regression models assessing independent associations between anthropometric data and cardiovascular risk factors, and vascular parameters.

Dependent variable	N	R^2	Model P-value
Independent variables	*B*	*CI95*	*P-value*
Carotid IMT [µm]	177	0.25	<0.001
Constant	*103.0*	*-13.0;218.9*	*0.081*
Age [years]	***4.68***	***2.43;6.91***	***<0.001***
BMI [kg/m^2]	***2.24***	***0.37;4.12***	***0.019***
Systolic BP [mmHg]	***0.85***	***0.15;1.55***	***0.017***
Hypertension medication [y/n]	*30.7*	*-3.06;64.5*	*0.074*
Brachial IMT [µm]	178	0.29	<0.001
Constant	*36.0*	*-10.1;82.1*	*0.125*
Age [yeras]	***1.70***	***0.84;2.56***	***<0.001***
BMI [kg/m^2]	***1.08***	***0.35;1.82***	***0.004***
Systolic BP [mmHg]	***0.36***	***0.08:0.63***	***0.011***
Hypertension medication [y/n]	***16.8***	***3.78;29.9***	***0.012***
Radial IMT [µm]	178	0.24	<0.001
Constant	23.4	-23.6;70.5	0.327
**Age [yeras]**	**1.79**	**0.88;2.71**	**<0.001**
BMI [kg/m^2]	0.18	-0.50;0.86	0.609
**Systolic BP [mmHg]**	**0.47**	**0.17:0.76**	**0.002**
**Hypertensive treatment [y/n]**	**19.0**	**5.4;32.5**	**0.006**
Carotid DC [%/10mmHg]	159	0.17	<0.001
Constant	*6.05*	*4.35;7.75*	*<0.001*
Age [yeras]	***-0.05***	***-0.10;-0.01***	***0.012***
BMI [kg/m^2]	*-0.01*	*-0.04;0.03*	*0.690*
Metabolic Syndrome [y/n]	***-0.80***	***-1.27;-0.33***	***0.001***
Smoking >10 pack years [y/n]	***-0.49***	***-0.95;-0.04***	***0.035***
Carotid β-stiffness index	159	0.09	0.015
Constant	*2.25*	*0.45;4.05*	*0.015*
Age [yeras]	*0.02*	*-0.01;0.06*	*0.240*
BMI [kg/m^2]	*0.00*	*-0.03;0.03*	*0.998*
Metabolic Syndrome [y/n]	***0.50***	***0.09;0.90***	***0.015***
Smoking >10 pack years [y/n]	***0.42***	***0.03;0.81***	***0.036***
Carotid-Femoral PWV [m/s]	189	0.21	<0.001
Constant	*4.16*	*2.28;6.03*	*<0.001*
Age [years]	***0.07***	***0.04.0.11***	***<0.001***
BMI [kg/m^2]	*-0.02*	*-0.05;0.02*	*0.384*
Waist-hip ratio > 0.85 [y/n]	***0.61***	***0.23;1.00***	***0.002***
Triglycerides [mmol/l]	***0.48***	***0.17;0.80***	***0.003***
Hypertension [y/n]	***0.62***	***0.10;1.13***	***0.018***

BMI – Body mass index; BP – Blood pressure; DC – Distensibility coefficient; IMT – Intima media thickness; PWV – Pulse wave velocity; PY – Pack years.
